# Development and Content Validation of Entrustable Professional Activities for Community Pharmacy Practice Experiences in Brazil

**DOI:** 10.1111/tct.70495

**Published:** 2026-08-10

**Authors:** Luciana Flavia de Almeida Romani, Marina Guimaraes Lima

**Affiliations:** ^1^ Post Graduation Programme in Medicines and Pharmaceutical Services Federal University of Minas Gerais Belo Horizonte Minas Gerais Brazil; ^2^ Department of Social Pharmacy, Faculty of Pharmacy Federal University of Minas Gerais Belo Horizonte Minas Gerais Brazil

**Keywords:** community pharmacy services, entrustable professional activities, EQual rubric, experiential learning, pharmaceutical education

## Abstract

**Introduction:**

Entrustable Professional Activities (EPAs) have been adopted in community pharmacy practice experiences, but there are no studies describing the development and validation of EPAs in contexts before their implementation. This study aimed to develop and validate the content of EPAs for community pharmacy practice experiences in Brazil.

**Methods:**

A multi‐stage research approach based on established theoretical frameworks for EPA development was employed. Stages included: (1) team formation and preparation; (2) identification and adaptation of a validated quality assessment tool (Queen's EPA Quality—EQual) for the Brazilian context; (3) drafting EPAs by mapping Brazilian national competencies to established EPAs of the United States of America, and adapting them linguistically and contextually; (4) two rounds of expert content validation utilizing the EQual rubric in Portuguese.

**Results:**

Seven experts participated in the first round of evaluation; six contributed to the second. Initial assessment of 11 EPAs revealed only two EPAs surpassing all domain‐specific and overall quality cut‐offs in the EQual tool. The initial list of EPAs was adjusted and sent back for a second round of evaluation. The final list comprised three required EPAs scoring above the EQual rubric's overall and domain‐specific cut‐offs.

**Conclusion:**

This research proposed a list of EPAs for community pharmacy practice experiences to be adopted for the first time, offering a framework to standardize teaching, inform preceptor training, and reliably assess student competency development.

## 1 Introduction

Entrustable Professional Activities (EPAs) were proposed by ten Cate [1] in 2005 to refocus competency‐based medical education. EPAs are essential tasks of one profession that can be entrusted to learners once they have attained the required competencies to perform them [1]. The EPAs' framework provides explicit expectations for clinical tasks that students are required to perform and makes assessments of students' competencies more meaningful [2].

The EPAs have been adopted in medical education since the 2010s, especially in the United States of America (USA) [3]. Although originally conceptualized for medical education, the EPAs' framework has also been adopted in the education of other health professions, including nursing [4], dentistry [5], and pharmacy [6]. Authors in this field have recommended using validated tools to assess quality of EPAs during the development stage [2], such as the Queen's EPA Quality (EQual) [7]. Few studies appraised the quality of EPAs through an EQual rubric in pharmaceutical education in the USA and Australia [6, 8], but they were carried out after the EPAs were incorporated into curricula. It is important to evaluate the validity of the EPAs before committing to them [2, 9].

*It is important to evaluate the validity of the EPAs before committing to them*.

The pharmacy profession has transitioned from a medication‐centered to a patient‐centered model, with progressive incorporation of clinical activities among its tasks [10]. The main workplace for pharmacists worldwide is the community pharmacy, which provides medicine‐related services and is widely accessed by the population [11]. Practice experiences in community pharmacy settings are common in pharmaceutical education to develop students' competencies [12]. Despite their learning outcomes, there are barriers in community pharmacy practice experiences, including a disconnect between theoretical knowledge and practice [13, 14], and insufficient exposure to the different processes and roles of a community pharmacist [15, 16]. The EPAs have been adopted in practice experiences by pharmacy programs [17]. In one study conducted in the USA, the researchers developed and obtained consensus among preceptors about the EPAs students should perform in community pharmacy practice experiences [18]. However, this study included preceptors experienced with the EPAs' framework and did not provide insights into how EPAs could be developed in contexts prior to their implementation.

This study aimed to develop and validate the content of EPAs for community pharmacy practice experiences in Brazil. In this country, pharmacy programs are not yet experienced in the EPAs' framework.

## 2 Methods

This was a study about the development and content validation of EPAs for community pharmacy practice experiences. The approach adopted by this research was based on the theoretical framework for designing EPAs in scientific literature [2, 19]. The following stages were included:

1. Team formation, preparation, and definition of a tool to check the quality of EPAs;
2. Development of an initial draft of EPAs;
3. Content validation of EPAs among experts.

Ethical approval was obtained from the Ethics Committee of the Federal University of Minas Gerais (Protocol 6.853.744 May 2024, CAAE 79364324.0.0000.5149). The stages of this study were described in the subsections bellow.

### 2.1 Team Formation, Preparation, and Definition of a Tool to Check the Quality of EPAs

The team of researchers consisted of the two authors of this study. They expanded their expertise on the EPAs' framework and established a shared understanding of the purpose of the EPAs. In the stage of team preparation, the researchers identified the EQual as the most used tool to check the quality of EPAs in the literature [2, 6, 9]. The researchers translated the items of the EQual tool to the Portuguese language, adapting them to the context of the pharmacy practice in Brazil. As EQual tool was originally designed for medical education, the word “physician” was changed to “pharmacist”. The researchers produced one instructional material about the EPA and EQual rubric in Portuguese.

### 2.2 Development of an Initial Draft of EPAs

The researcher team explored existing EPAs in pharmaceutical education and identified validated core EPAs in the USA [20, 21] and Australia [6]. The researchers considered the core EPAs for new pharmacy graduates of the USA more suitable for the context of pharmacy practice in Brazil.

The two authors mapped the 22 competencies of the Brazilian Standard Guidelines for Pharmaceutical Education related to community pharmacy practice [22] to the 13 EPAs for new pharmacy graduates in the USA [20]. The text of the 11 EPAs from the USA selected was translated into the Portuguese language and adapted to the Brazilian context of community pharmacy practice, using terminologies adopted in a publication of the Brazilian Pharmacy Federal Council [23]. The authors designed a list of 11 EPAs, with their description and supporting tasks, which was assessed and revised by two faculty members of the same institution as the researchers. This stage was held for 5 weeks, from October to November 2024.

### 2.3 Content Validation of EPAs Among Experts

In the content validation, experts and possible end users of EPAs for community pharmacy practice experience programs were invited to appraise critically the quality of EPAs and contribute to the achievement of a consensus on them. The experts chosen were pharmacists, preceptors involved in community pharmacy practice experience programs, research‐skilled, and from different regions of Brazil. They were identified through a purposive sample. The experts who agreed to participate in the study were provided with resources to assist them in understanding the EPA construct and completing the EQual rubric, including a guide and a video designed by the authors. In the first round, they answered an electronic survey on the Google Forms platform. The survey page included a consent form, participant demographics, and each subsequent page included each EPA next to the 14 items of the EQual rubric. Each question within the EQual rubric has five responses with descriptive anchors scoring between 1 and 5 [7]. The survey also had three prompts asking if and why the EPA requires revision, along with a free‐text opportunity to respond to and recommend revisions. The data of this first round of the survey were collected from December 2024 to March 2025 and inserted into a Microsoft Excel sheet. Averages for EQual rubric scores were calculated for each of the 11 EPAs, overall and for each of the three domains (discrete units of work; entrustable, essential, and important task of the profession; and EPAs' curricular role). Participant demographics and expert suggestions were summarized. To measure the internal consistency of the translated and adapted EQual rubric, the Cronbach's alpha coefficient was calculated. Each EPA that received an overall average score below the cut‐off of 4.07 or any of the domain‐specific cut‐offs (4.17, 4.00, and 4.00 for discrete units of work; entrustable, essential, and important task of the profession; and EPA's curricular role, respectively) was revised. Based on the suggestions of experts and an analysis of the reasons for each EPA scoring below the cut‐off, the authors revised the list of EPAs. Adjustments were made in the nine EPAs identified as requiring revisions, and a list of four revised EPAs was sent to the experts for the second round of evaluation. The data of this second round of the survey were collected from March to May 2025. The EQual rubric scores were calculated in the same manner as the first round, and expert suggestions were summarized again. Microsoft Excel was used in all statistical analyses. The researchers performed adjustments and wrote the final list of EPAs and their supporting tasks, based on the analysis of the data collected in the second round of assessment.

## 3 Results

In the stage of content validation of EPAs, 19 experts were identified by the researchers, and seven completed the first round of the EQual rubric evaluation. Six experts were faculty members. Four participants held a PhD title, and three held a master's degree. Their mean age was 42 years, with five females and two males.

In the first round of the EQual rubric evaluation, 11 EPAs were assessed by seven experts. The questionnaire with the EQual rubric translated and adapted to the Brazilian pharmacy practice showed a high internal consistency (the Cronbach's alpha coefficient = 0.95). Five of 11 EPAs were below the overall cut‐off score of 4.07 in the first round of the evaluation (Table 1). Regarding the domain discrete units of work, nine of 11 EPAs did not reach the cut‐off score of 4.17. Three of the 11 EPAs were also below the cut‐off score of 4.00 on the domain entrustable, essential, and important tasks of the profession, and one on the domain EPA's curricular role.

**TABLE 1:** Overall and domain‐specific average scores for the 11 EPAs for community pharmacy practice experience programs in Brazil, first round of the EQual rubric evaluation (*N* = 7).

EPA number and title	Average score in domain discrete units of work	Average score in domain entrustable, essential, and important task of the profession	Average score in domain EPA's curricular role	Overall score average
EPA 1—Collect information necessary to identify a patient's medication‐related problems and health‐related needs	4.19	4.25	4.11	4.18
EPA 2—Assess collected information to determine a patient's medication‐related problems and health‐related needs	**3.76** *	4.50	4.57	4.28
EPA 3—Create and implement a care plan in collaboration with the patient, others trusted by the patient, and other health professionals	**4.00** *	4.75	4.50	4.42
EPA 4—Monitor the effectiveness and safety of the interventions through follow‐up interviews with patients	**3.83** *	4.25	4.39	4.16
EPA 5—Contribute patient‐specific medication‐related expertise as part of an interprofessional care team through meetings, lectures, reports, and other communication types	**3.45** *	4.25	**3.93** ***	**3.88** ****
EPA 6—Answer medication‐related questions using scientific literature	**3.76** *	4.21	4.32	4.10
EPA 7—Deliver medication or health‐related education to patients on individual and collective levels	**3.81** *	**3.54** **	4.14	**3.83** ****
EPA 8—Report adverse drug events, medication errors, and drug quality deviations	**4.02** *	**3.68** **	4.11	**3.94** ****
EPA 9—Identify populations at risk for prevalent diseases and preventable adverse medication outcomes, from the data collection to the definition of risk situations and interventions	**3.12** *	4.04	4.21	**3.79** ****
EPA10—Perform the technical, administrative, and supporting operations of a pharmacy, from the initial planning of management of stock, processes, and human resources to the final assessment and needed adjustments to assure the efficient operation of a pharmacy	**3.26** *	**3.86** **	4.46	**3.86** ****
EPA 11—Dispense medicines	4.31	4.25	4.64	4.40

* Score below the cut‐off for the domain discrete units of work (4.17).

** Score below the cut‐off for the domain entrustable, essential, and important task of the profession (4.00).

*** Score below the cut‐off for the domain EPA's curricular role (4.00).

**** Overall score below the cut‐off (4.07).

The experts made suggestions suitable for improving the quality of the EPAs. One expert recommended excluding EPA 4 (Monitor the effectiveness and safety of the interventions) because it overlapped with EPA 1 (Collect information), EPA 2 (Assess collected information), and EPA 3 (Create and implement a care plan). One expert suggested excluding EPA 9 (Identify populations at risk) due to its overlap with other EPAs. Two experts considered EPA 10 (Perform the technical, administrative, and supporting operations) too broad and suggested separating it into two EPAs: one focused on stock management and another on management of processes and human resources. The participants also made suggestions for the improvement of the list of supporting tasks of EPA 4 (monitor the effectiveness and safety of the interventions), EPA 5 (contribute patient‐specific medication‐related expertise), and EPA 11 (dispensing medicines).

The nine EPAs identified as requiring revisions were adjusted by the researchers based on findings of the first round of the evaluation. The researchers removed EPA 4 (Monitor the effectiveness and safety of the interventions) and EPA 9 (Identify populations at risk), as suggested by the experts. They also excluded EPA 5 (Contribute patient‐specific medication‐related expertise) and EPA 8 (Report adverse drug events) due to their poor score and overlapping with other EPAs, then transferred their supporting tasks to other EPAs. The researchers merged EPA 6 (Answer medication‐related questions) and EPA 7 (Deliver medication or health‐related education) into one EPA titled “Disseminate rational drug use information to the population through instructional materials, lectures, educational activities, and answering medication related questions” because EPA 7 scored below the overall cut‐off and was overlapped with EPA 6. To minimize the overlapping, the researchers merged EPA 2 (Assess collected information) and EPA 3 (Create and implement a care plan) into one EPA titled “Create, implement, and monitor a pharmaceutical care plan”. As suggested by the experts, the researchers separated EPA 10 (Perform the technical, administrative, and supporting operations) into two EPAs: “Perform stock management by technical and scientific standards and the current pharmaceutical legislation” and “Perform management of the processes and the team to assure patient safety and efficient operation of a pharmacy”. Considering these changes, a list of four revised EPAs was presented back to the experts.

Six of seven experts responded to the second round of the rubric evaluation and appraised the four revised EPAs. All four revised EPAs were above the overall cut‐off score of 4.07, but the EPA about the management of processes and the pharmacy team (Table 2). Four experts suggested improvements for the text of the supporting tasks of these four revised EPAs. One expert considered the EPA “Create, implement, and monitor a pharmaceutical care plan” too broad and suggested separating it into two EPAs: one focused on clinical assessment and another on care plan implementation. The researchers decided to add to the final list the three EPAs that scored above the overall and domain‐specific cut‐offs. Other EPAs that did not reach the overall and domain‐specific cut‐offs were included as elective. Table S1 showed a list of elective EPAs. Table 3 presents the final list of three required EPAs for Brazilian community pharmacy practice experience programs in undergraduate pharmaceutical education.

**TABLE 2:** Overall and domain‐specific average scores for the four revised EPAs, second round of the EQual rubric evaluation (*N* = 6).

EPA title	Average score in domain discrete units of work	Average score in domain entrustable, essential, and important task of the profession	Average score in domain EPA's curricular role	Overall score average
Create, implement, and monitor a pharmaceutical care plan	**3.67** *	4.54	4.54	4.25
Disseminate rational drug use information to the population through instructional materials, lectures, educational activities, and answering medication‐related questions	**3.97** *	4.08	4.37	4.14
Perform stock management by technical and scientific standards and the current pharmaceutical legislation	4.50	4.21	4.12	4.28
Perform management of the processes and the team to assure patient safety and efficient operation of a pharmacy	**3.83** *	**3.71** *	4.25	**3.93** ***

* Score below the cut‐off for the domain discrete units of work (4.17).

** Score below the cut‐off for the domain entrustable, essential, and important task of the profession (4.00).

*** Overall score below the cut‐off (4.07).

**TABLE 3:** Final list of three required EPAs for Brazilian community pharmacy practice experience programs in undergraduate pharmaceutical education.

EPA title	Supporting tasks
Collect information necessary to identify a patient's medication‐related problems and health‐related needs	Patient welcoming Conduct a pharmaceutical anamnesis, interviewing the patient or caregiver to collect clinical data Collect the patient's clinical data from medical records or information systems Perform clinical‐laboratory exams or measure physiological parameters, such as blood pressure and body temperature Conduct rapid tests according to the manufacturer's instructions, ensuring accuracy of results
Perform stock management by technical and scientific standards and the current pharmaceutical legislation	Carry out scheduling of medicines using methods recommended by scientific and technical literature Receive and check medicines and supplies by institutional standards Conduct an inventory of medicines according to institutional regulations Record data on the entry and exit of medicines Monitor stock indicators and parameters to ensure the necessary supply of medications and supplies, as well as compliance with current legislation Check the storage conditions of medicines to preserve their quality
Dispense medicines	Patient welcoming Evaluate prescriptions regarding legal and therapeutic aspects Collect additional patient information and identify potential medication‐related problems Provide pharmacological and non‐pharmacological guidance Supply the patient with the necessary quantity of medicines Carry out administrative control of dispensed medications Carry out electronic record‐keeping of controlled products in the National System for the Management of Controlled Products Compound and ensure the quality of medicines, if necessary Administer injectables and vaccines using correct techniques for each route of administration (intramuscular, subcutaneous, intradermal, intravenous) and record all relevant information about the procedure, including the name of the medication, dose, route of administration, date, and time of administration; and comply with the current legislation

## 4 Discussion

This study aimed at developing and validating the content of EPAs for community pharmacy practice experience programs in Brazilian pharmaceutical undergraduate education. After two rounds of assessment of quality of EPAs by experts, three EPAs scored above the overall and domain‐specific cut‐offs of the EQual tool and were included in the final list of required EPAs for community pharmacy practice experiences.

To the authors' knowledge, this is the first time that EPAs have been developed and validated for community pharmacy practice experience programs before their implementation. However, a similar study was conducted in the USA post‐EPAs' implementation [24]. In this research, an approach based on the theoretical framework for designing EPAs was adopted [2, 19] to ensure their quality and usefulness as an educational tool that assists the learner. The use of one validated rubric to assess the quality of EPAs by their end users (preceptors) may assist in future implementation and acceptability of EPAs in the workplace. Some studies found that the involvement of stakeholders in an EPA development process resulted in greater acceptance of the EPAs [24, 25].

Some challenges were observed during the development and content validation processes. With EPA being a new conceptual framework in Brazilian pharmaceutical education, it would be challenging for pharmacist preceptors to understand the EPA construct and assess the quality of EPAs developed. This sort of challenge was also observed in a study of EPA development in nursing education, in which preceptors and faculty members dispensed additional time and effort to be familiarized with the EPAs' framework [4]. In the current research, the experts selected by the researchers were research‐skilled and were provided with instructional materials to prepare them for the assessment of the quality of EPAs. These approaches can be adopted by educators who intend to design EPAs for the first time to qualify practice experiences in health professions education.

In the first round of EQual assessment of EPAs developed, most of them scored below the discrete units of work cut‐off. This problem was also observed in other studies of quality assessment of EPA through EQual in pharmaceutical education [6, 8]. In a study of appraisal of quality of core EPAs for new pharmacy graduates in the USA, the experts commented that the EPAs about clinical assessment and implementation of a care plan overlap and are not independent activities [8]. In the current study, the EPA about collaboration with an interprofessional care team was not considered as an independent, measurable, and observable task. The experts also judged that this EPA did not require the application of knowledge, skills, and attitudes acquired through training. Some studies in medical and pharmaceutical education showed similar findings [8, 9]. According to an evaluation of the core EPAs for entering residency in medical education in the USA, the experts provided low scores for the EPA “Collaborate as a member of an interprofessional team”, arguing that this EPA is not a discrete unit of work and suggesting the incorporation of it into other EPAs [9]. The different contributions that a pharmacist can make within a team, such as meetings, lectures, and reports, may support tasks of other EPAs about care plan implementation and medication‐related education. For example, in the current study, “Discuss patients' clinical cases with other healthcare professionals” was added as a supporting task of the EPA “Create and implement a care plan in collaboration with the patient, others trusted by the patient, and other health professionals”.

According to a scoping review about practice experience in community pharmacies [12], among the activities most performed by the students were collecting information to identify a patient's medication‐related problems and dispensing medicines. Not coincidentally, these two were the only EPAs that scored above the cut‐off on all domains in the current study. These findings are aligned with the activities traditionally performed by community pharmacists throughout the world, focused on dispensing medicines [26]. Despite a trend of increase in clinical services in community pharmacies, they are not widely performed due to barriers of this practice setting, such as organizational culture and time constraints [27]. The implementation of EPAs in pharmacy education can contribute to standardizing the clinical practice of future pharmacists.

The experts established a consensus on three EPAs for community pharmacy practice experiences in Brazilian pharmaceutical undergraduate education: collecting patient's information, performing stock management, and dispensing medicines. These EPAs reflect the activities most frequent in community pharmacy practice experiences worldwide [12]. The scientific literature presents heterogeneity in the number of EPAs adopted in clinical training. In a study about uptake of EPAs in Dutch postgraduate medical programs, the number of EPAs ranged from 12 to 120 per program [28]. In the general practice program—characterized by its holistic role in care and its broad variation of patients—there was a smaller number of EPAs formulated more generally [28]. A survey about medical undergraduates' perceptions of workplace‐based assessments (WBAs) using EPAs found that most students did not think WBAs were valuable and reported no feedback from preceptors in a timely fashion [29]. Based on these findings, the researchers of this study proposed for future classes WBAs aligned with only three EPAs out of the 13 core EPAs recommended by the Association of American Medical Colleges in the USA [29]. The pharmacist's practice in community pharmacy is holistic as medical general practice, with a high variety in patient population. The workplace environment of community pharmacy practice experiences can face the same challenges observed in undergraduate medical education, with preceptors not available for feedback on many EPAs due to time constraints. Considering this, the number of three required EPAs can be feasible for community pharmacy practice experiences.

The current study has some limitations. The experts who participated in the content validation did not have experience in using EPAs because they had not been implemented in Brazilian pharmaceutical education. The EQual rubric was originally designed for EPAs in medical education and therefore uses medical terminology. Pharmaceutical non‐clinical tasks such as stock management may have been underscored because they are not an essential task of the medical profession. Another limitation was the small number of participants, but this was observed in other studies on quality assessment of EPAs in medical and pharmaceutical education [6, 8, 9].

## 5 Conclusions

For the first time, a list of EPAs was developed and had its content validated for community pharmacy practice experience programs previously to their implementation. These EPAs may provide a standardized and reliable approach to designing teaching activities, inform preceptor training, and enhance and assess the development of competencies by the students. This research described in detail the stages of EPA development and content validation and presented a list of supporting tasks for EPAs. These findings can inform faculty members and preceptors who intend to design new EPAs in health professional education.

## Author Contributions

**Luciana Flavia de Almeida Romani:** conceptualization, methodology, formal analysis, writing – original draft, data curation, investigation, project administration, writing – review and editing. **Marina Guimaraes Lima:** conceptualization, methodology, writing – original draft, writing – review and editing, supervision, project administration, data curation, formal analysis, investigation.

## Funding

This work was supported by Coordenação de Aperfeiçoamento de Pessoal de Nível Superior (CAPES).

## Ethics Statement

Ethical approval was obtained from the Ethics Committee of the Federal University of Minas Gerais (Protocol 6.853.744 May 2024, CAAE 79364324.0.0000.5149). Informed consent was obtained from each participant before they were submitted to any study procedure.

## Conflicts of Interest

The authors declare no conflicts of interest.

## Supporting information


**Table S1:** List of elective EPAs for Brazilian community pharmacy practice experience programs in undergraduate pharmaceutical education

## Data Availability

Research data are not shared.
