# Supplementary material for: Development and Content Validation of Entrustable Professional Activities for Community Pharmacy Practice Experiences in Brazil
Source: Clin Teach. 2026 Aug 10;23(5):e70495. doi: 10.1111/tct.70495 (PMC13457503; doi:10.1111/tct.70495)
Supplement: Supplementary file 1 [file TCT-23-e70495-s001.docx]

**TABLE S1**- List of elective EPAs for Brazilian community pharmacy practice experience programs in undergraduate pharmaceutical education

| EPA title | Supporting tasks |
| --- | --- |
| Assess collected information to determine a patient’s medication-related problems and health-related needs | - Identify individuals at risk for health problems and preventable adverse events that have not yet been effectively diagnosed or treated - Assess whether the patient needs to be referred to another healthcare professional - Interpret clinical data collected from the patient, including results from clinical and laboratory exams - Review the patient’s pharmacotherapy, prepare a list of identified problems, and provide recommendations to the patient or healthcare team - Seek and interpret reliable sources of information in scientific literature - Identify adverse drug events or medication errors - Document and report adverse drug events or medication errors to the National Pharmacovigilance System, according to institutional procedures |
| Create and implement a care plan in collaboration with the patient, others trusted by the patient, and other health professionals | - Define therapeutic goals in collaboration with the patient, their caregiver, and other healthcare professionals - Document proposed interventions to prevent or resolve medication-related problems - Discuss patients’ clinical cases with other healthcare professionals - Prescribe medicines, if necessary - Guide the patient regarding the proposed interventions, employing educational strategies to increase their active participation, understanding, and adherence to treatment - Define parameters and the frequency for monitoring the outcomes of the care plan |
| Monitor the effectiveness and safety of the interventions through follow-up interviews with patients | - Evaluate the results of the care plan upon the patient’s return to the pharmacy by reviewing exam results (objective parameters) or by the patient’s report/experience (subjective parameters), and adjust the plan if necessary |
| Disseminate rational drug use information to the population through instructional materials, lectures, educational activities, and answering medication-related questions | - Seek and interpret reliable sources of information in scientific literature - Identify the learning needs of the population related to the rational use of medicines - Provide guidance and answer medication-related questions using scientific literature - Disseminate rational drug use information to the population through instructional materials, lectures, educational activities, both in-person and online, adapting the language to the level of understanding of the patient or health professional |
| Perform management of the processes and the team to assure patient safety and efficient operation of a pharmacy | - Establish processes, protocols, and procedures that contribute to reducing medication errors and promoting a patient safety culture. - Establish processes, protocols, and procedures that contribute to the efficient operation of the pharmacy. - Implement and monitor intervention plans to ensure the quality of pharmacy processes and services. - Conduct team training on topics related to the rational use of medications and pharmacy procedures. - Supervise and monitor the team regarding pharmacy procedures. - Set up performance evaluations with feedback for workers. - Establish mechanisms for shared decision-making regarding the patient safety culture and the efficient operation of the pharmacy. |
